# Interrater variability of ML-based CT-FFR during TAVR-planning: influence of image quality and coronary artery calcifications

**DOI:** 10.3389/fcvm.2023.1301619

**Published:** 2023-12-21

**Authors:** Robin F. Gohmann, Adrian Schug, Konrad Pawelka, Patrick Seitz, Nicolas Majunke, Hamza El Hadi, Linda Heiser, Katharina Renatus, Steffen Desch, Sergey Leontyev, Thilo Noack, Philipp Kiefer, Christian Krieghoff, Christian Lücke, Sebastian Ebel, Michael A. Borger, Holger Thiele, Christoph Panknin, Mohamed Abdel-Wahab, Matthias Horn, Matthias Gutberlet

**Affiliations:** ^1^ Department of Diagnostic and Interventional Radiology, Heart Center Leipzig, Leipzig, Germany; ^2^ Medical Faculty, University of Leipzig, Leipzig, Germany; ^3^ Department of Cardiology, Heart Center Leipzig, University of Leipzig, Leipzig, Germany; ^4^ Department of Cardiac Surgery, Heart Center Leipzig, University of Leipzig, Leipzig, Germany; ^5^ Helios Health Institute, Leipzig, Germany; ^6^ Siemens Healthcare GmbH, Erlangen, Germany; ^7^ Institute for Medical Informatics, Statistics and Epidemiology (IMISE), University of Leipzig, Leipzig, Germany

**Keywords:** aortic stenosis, computed tomography coronary angiography, coronary angiography, coronary artery disease, transcatheter aortic valve implantation, diagnostic accuracy, machine learning, computed tomography fractional flow reserve

## Abstract

**Objective:**

To compare machine learning (ML)-based CT-derived fractional flow reserve (CT-FFR) in patients before transcatheter aortic valve replacement (TAVR) by observers with differing training and to assess influencing factors.

**Background:**

Coronary computed tomography angiography (cCTA) can effectively exclude CAD, e.g. prior to TAVR, but remains limited by its specificity. CT-FFR may mitigate this limitation also in patients prior to TAVR. While a high reliability of CT-FFR is presumed, little is known about the reproducibility of ML-based CT-FFR.

**Methods:**

Consecutive patients with obstructive CAD on cCTA were evaluated with ML-based CT-FFR by two observers. Categorization into hemodynamically significant CAD was compared against invasive coronary angiography. The influence of image quality and coronary artery calcium score (CAC) was examined.

**Results:**

CT-FFR was successfully performed on 214/272 examinations by both observers. The median difference of CT-FFR between both observers was −0.05(−0.12-0.02) (*p* < 0.001). Differences showed an inverse correlation to the absolute CT-FFR values. Categorization into CAD was different in 37/214 examinations, resulting in net recategorization of Δ13 (13/214) examinations and a difference in accuracy of Δ6.1%. On patient level, correlation of absolute and categorized values was substantial (0.567 and 0.570, *p* < 0.001). Categorization into CAD showed no correlation to image quality or CAC (*p* > 0.13).

**Conclusion:**

Differences between CT-FFR values increased in values below the cut-off, having little clinical impact. Categorization into CAD differed in several patients, but ultimately only had a moderate influence on diagnostic accuracy. This was independent of image quality or CAC.

## 1. Introduction

Patients evaluated to be treated with transcatheter aortic valve replacement (TAVR) are generally elderly and have a high prevalence of coronary artery disease (CAD) (1–3). CAD is recommended to be excluded and if needed to be treated before the procedure (3–7). Coronary computed tomography angiography (cCTA) is the first line diagnostic tool for the exclusion of CAD in other patient groups (7) and its high negative predictive value (NPV) is known to be preserved also in patient before TAVR (3, 6, 8). Thus, its use is increasingly recognized as part of the standard CT evaluation protocol for TAVR-planning (3–8). However, cCTA remains limited by its low specificity, particularly in this patient group. CT-derived fractional flow reserve (CT-FFR) has been described as a promising tool to mitigate this limitation by non-invasively predicting hemodynamic relevance (9–11) also in patients prior to TAVR (12–16).

Machine learning (ML)-based CT-FFR is a computationally less demanding approach, which makes on-site computation of CT-FFR feasible on standard workstations and is known to correlate well with the more conventional computational fluid dynamics (CFD) approach (17). As opposed to the commercial off-site approaches, where the exact segmentation process is unknown, the user himself performs the segmentation in ML-based CT-FFR. While a high reliability of segmentation is presumed, the significance of the segmentation process and observer experience on the reliability of CT-FFR has not been well examined (18, 19), with no systematic analysis as of now.

In this study, we systematically compared the ML-based CT-FFR measurements carried out by two observers with differing expertise, on segment, vessel and patient level in a large group of patients before TAVR. Furthermore, we analyzed the frequency of conflicting categorizations and the influence of image quality and coronary artery calcium score (CAC).

## 2. Material and methods

### 2.1. Study design and patient population

**Figure 1:**
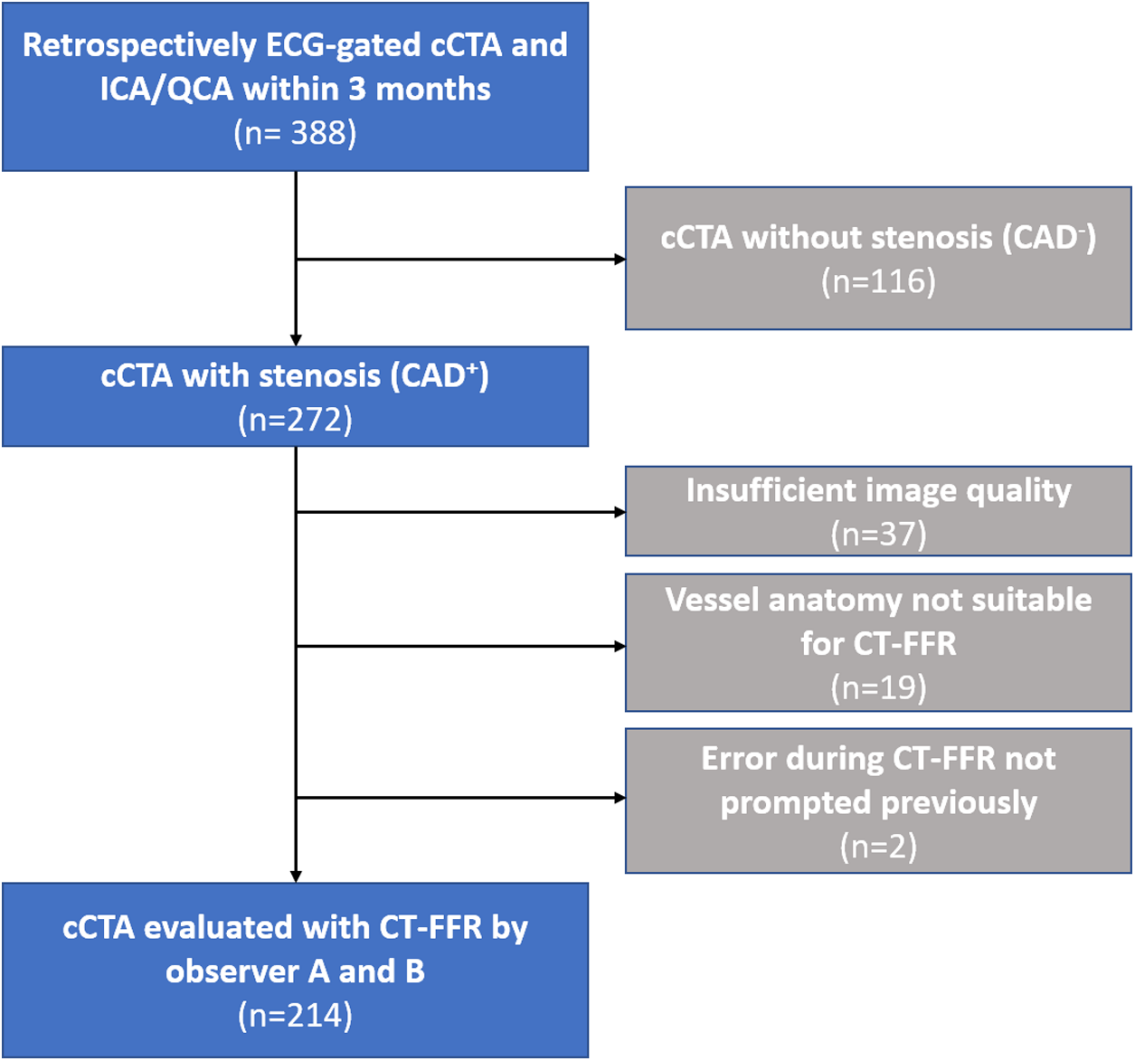
Flowchart of the study population. Flowchart of the study population and reasons for exclusion. CAD, coronary artery disease; cCTA, coronary computed tomography angiography; CT-FFR, CT-derived-fractional-flow-reserve; ECG, electrocardiogram; ICA, invasive coronary angiography; QCA, quantitative coronary angiography.

The patient population and study design have previously been reported on (8, 13). In short, consecutive examinations with retrospectively ECG-gated CT for TAVR-planning over a period of 7 months were screened. Only patients having undergone invasive coronary angiography (ICA) within 3 months of CT were considered for the current analysis. Of the 388 patients, 272 had at least one coronary stenosis (≥50%) on cCTA being of interest for CT-FFR evaluation (Figure 1).

### 2.2. CT acquisition

The scan protocol has previously been described in detail (8). Briefly, a retrospectively ECG-gated helical CT of the heart was performed from caudal to cranial, immediately followed by a high-pitch helical CT in the opposite direction for depiction of the aorta and iliofemoral access route using a single bolus of 70 ml iodinated contrast medium. All patients were examined with the same scanner (Somatom Definition Flash; Siemens). No beta blockers or nitrates were given. The ECG-gated scan of the heart was used for computation of the ML-based CT-FFR.

### 2.3. cCTA and CT-FFR analysis

Coronary arteries were analyzed morphologically by segment according to the 18-segment model (20). When a stenosis of ≥50% diameter was identified on cCTA, CT-FFR values were obtained approximately 2 cm distal to the stenosis (21). The standard of reference was ICA with quantitative coronary analysis (QCA) with the same threshold and ≥70% for a secondary evaluation.

ML-based CT-FFR (cFFR version 3.2.0, Siemens; not commercially available) was performed by observer B on all examinations previously analyzed by observer A (13). The ML-based prototype used for this study has been described in detail before (13, 17, 22). The computationally less demanding process enabled on-site computation on a desktop workstation.

Per-segment interpretations were combined to form per-vessel and per-patient ratings, considering the respective worst segment (highest grade of stenosis; lowest CT-FFR value). CT-FFR values of ≤0.80 were considered as hemodynamically significant CAD (CAD^f+^) (23).

Both observers received the same instructions for segmentation and measurement of CT-FFR. Observer A had received several weeks of training in coronary artery imaging, including formal reading of cCTA and case discussions with correlation to ICA. Observer B only received comprehensive instructions on coronary artery segmentation and handling of the CT-FFR prototype at hand. Both measurements were taken within 18 months. The methods adopted for this study comply with the Guidelines for Reporting Reliability and Agreement Studies (GRRAS) (24).

### 2.4. Statistical analysis

Continuous variables are presented as median and [interquartile range (IQR)]. Differences between the two observers in CT-FFR values and evaluation times were assessed using the Wilcoxon signed-rank test. Interobserver agreement for CT-FFR values was evaluated using intra-class correlation (ICC) type ICC (1, 3) according to the convention proposed by Shrout and Fleiss (25). For interpretation of ICC coefficients, we followed the guidelines given by Cicchetti, which identify values <0.5 as a poor, 0.5–0.75 as moderate, 0.75–0.9 as a good, and >0.9 as an excellent correlation (26). Interobserver agreement with respect to categorization into hemodynamically significant CAD according to CT-FFR was assessed using Cohen's kappa and interpreted as proposed by Landis and Koch, which classifies correlation as follows: <0.2 slight, 0.2–0.4 fair, 0.4–0.6 moderate, 0.6–0.8 substantial, >0.8 almost perfect (27). Correlation between CT-FFR differences and covariates was calculated using Spearman's rank correlation (quantitative image quality measures and calcium burden) or Kendall's rank correlation (qualitative image quality). Correlation between mismatched coronary artery disease categorization and covariates was determined using the point-biserial correlation (quantitative image quality and CAC) or rank-biserial correlation (qualitative image quality). A *p* value of <0.05 was considered statistically significant. Statistical analysis was performed using R (version 4.1.2; R Foundation for Statistical Computing, Vienna, Austria).

## 3. Results

### 3.1. (Re)evaluation with ML-based CT-FFR

In total, 214 of the 272 examinations with signs of CAD on cCTA were successfully evaluated with ML-based CT-FFR by observer A and B (Figure 1). Two patients could not be reevaluated by observer B, because of an error with the prototype not prompted earlier. Reasons for initial exclusion were insufficient or borderline image quality hindering continuous segmentation of the coronary tree, and anatomical variants outside the model boundaries of the CT-FFR prototype (13). Evaluation time was significantly lower for observer A (observer A: 24 (18–32) min; observer B: 28 (22–35) min; *p* < 0.001).

Of the included patients 90 (42.1%) were female and the mean body mass index was 29.2 ± 5.5 kg/m^2^.

### 3.2. Differences in CT-FFR values

CT-FFR values were significantly different between observer A and B with the largest median differences on patient level (*n* = 214; −0.05[−0.12–0.02]; *p* < 0.001). Median differences on vessel level were also significantly different between the observers (left anterior descending artery [LAD] >left circumflex artery [LCX] >right coronary artery [RCA]). The LM had a much lower number of stenoses (*n* = 13) and thus could not be considered for analyses (Table 1).

**Table 1:** Interobserver variability of absolute CT-FFR values.

Level of observation		*n*	Difference	95% CI	*p*	ICC	95% CI	*p*
Patient		214	−0.05 (−0.12–0.02)	−0.065, −0.035	<0.001	0.567	0.469, 0.651	<0.001
Vessel	RCA	115	−0.03 (−0.09–0.03)	−0.055, −0.010	0.003	0.616	0.489, 0.718	<0.001
	LM	13	−0.03(−0.05–0.01)	−0.065, −0.005	0.04	0.427	−0.137, 0.782	0.064
	LAD	177	−0.04 (−0.12–0.05)	−0.06, −0.020	<0.001	0.558	0.447, 0.652	<0.001
	LCX	114	−0.04 (−0.09–0.03)	−0.055, −0.010	0.003	0.423	0.260, 0.563	<0.001

Values are median and (IQR) of the difference of CT-FFR values of the two observers. The first *p* value column (and 95% CI) corresponds to the difference of interobserver differences from zero while the second *p* value column (and 95% CI) corresponds to the difference of the ICC coefficient from zero. *P* values <0.05 were statistically significant. ICC, intra-class correlation coefficient; IQR, interquartile range; LAD, left anterior descending artery; LM, left main coronary artery; LCX, circumflex artery; RCA, right coronary artery.

**Figure 2:**
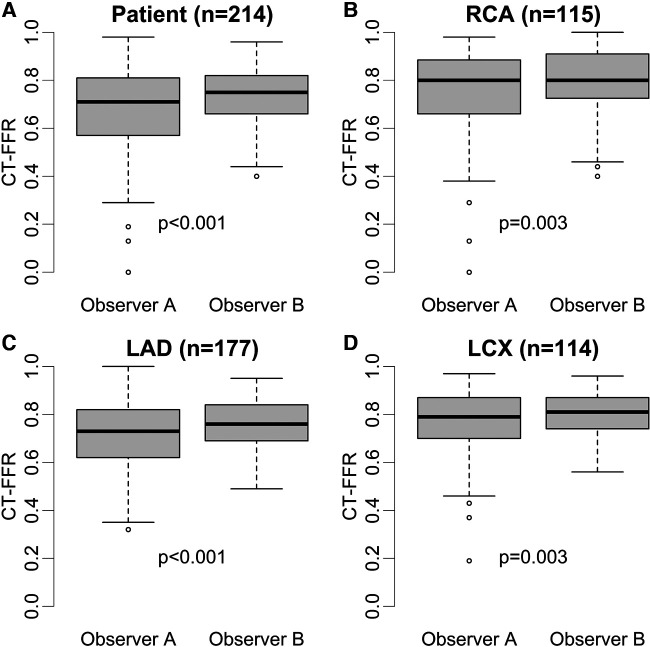
Distribution of absolute CT-FFR values. Box plots of CT-FFR values measured by two observers on patient (**A**) and vessel level (**B–D**). Observer B measured higher median CT-FFR values with a smaller interquartile range on patient and vessel level (*p *≤ 0.003) (**A–D**). The difference between both observers were larger on patient level (**A**) compared to vessel level (patient: 0.05> RCA: 0.05; LAD: 0.04; CX: 0.04) (**B–D**). Observer A shows more outliers, especially in RCA and LCX (**B,C**). CT-FFR, CT-derived fractional flow reserve; LAD, left anterior descending artery; LCX, left circumflex artery; RCA, right coronary artery.

**Figure 3:**
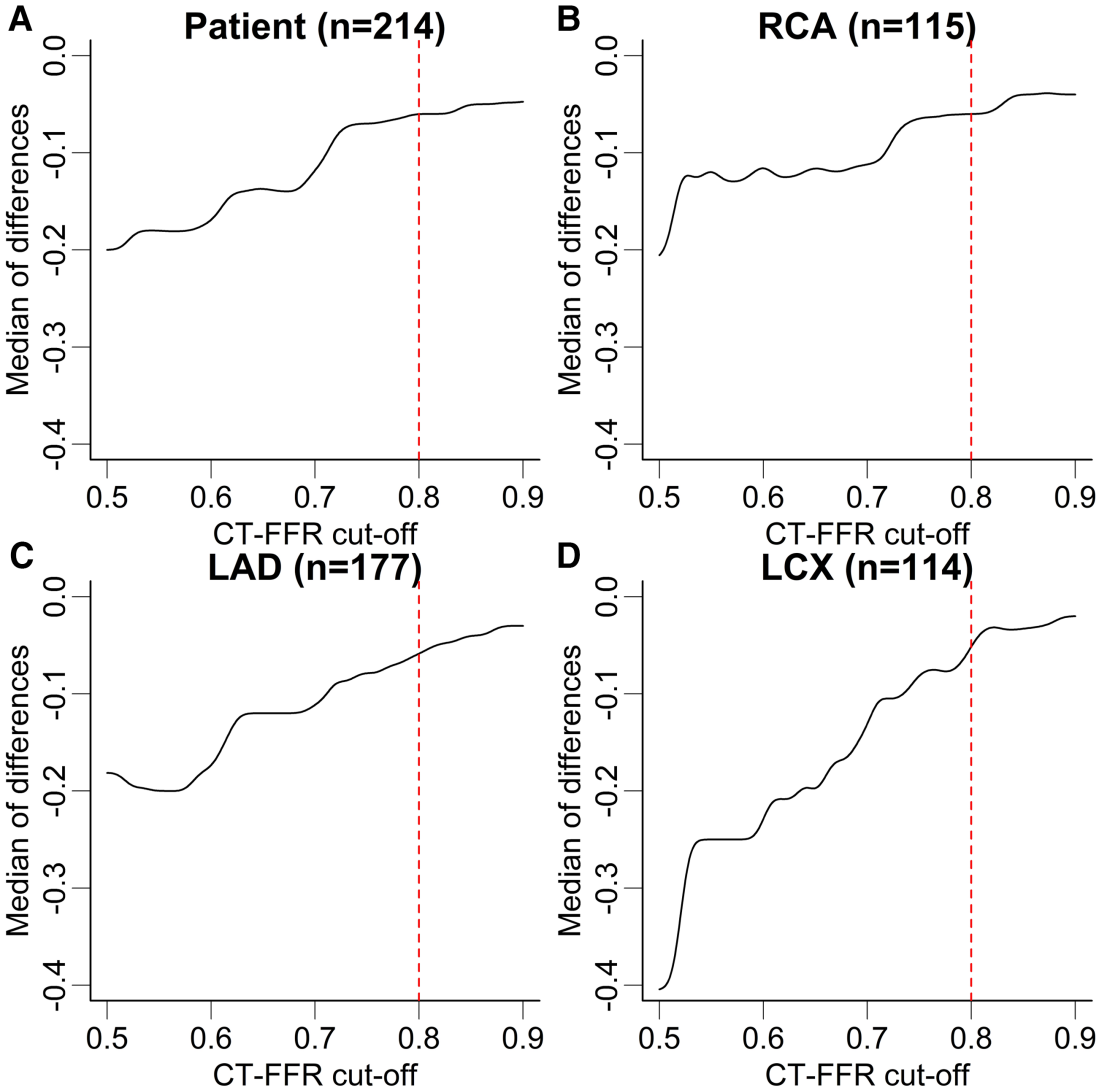
Median difference of CT-FFR values in dependence of absolute values. Median difference of CT-FFR values at different cut-offs between both observers at patient (**A**) and vessel level (**B–D**). The median difference of CT-FFR values is higher for low absolute values and lower for high values. Note, there is no discrete cut-off for all levels of observation, but differences are higher below the clinical cut-off CT-FFR ≤0.80. The dashed red lines correspond to the CT-FFR cut-off used to characterize hemodynamically significant CAD. The lines of the graph have been smoothed with a Gaussian filter to help avoid over-interpretation of small steps. CT-FFR, CT-derived fractional flow reserve; LAD, left anterior descending artery; LCX, left circumflex artery; RCA, right coronary artery.

Patients recategorized as false negative (FN) from true positive (TP) by either observer showed CT-FFR values closest to the cut-off of ≤0.80 (observer A: *n* = 17, 0.85 [0.83–0.87]; observer B: *n* = 28, 0.84 [0.82–0.89]). The distribution of CT-FFR values of both observers is shown in Figure 2. Observer A measured more outliers, particularly in the RCA and LCX. Discrepancies of CT-FFR values between the observers were smaller for high CT-FFR values, and larger for low values (Figure 3).

### 3.3. Interobserver variability

Analysis on patient level showed fair-good agreement between both observers (ICC coefficient: 0.567; *p* < 0.001). On vessel level, correlation between both observers was fair-good in the RCA and LAD. Agreement of measured values in the LCX was fair (Table 1).

Categorization into hemodynamically significant CAD according to CT-FFR correlated between both observers. On patient level, interobserver agreement was moderate-substantial (Cohen's kappa: 0.570; *p* < 0.001). Correlation in the RCA was moderate-substantial, in the LAD fair, and in the LCX fair (Table 2).

**Table 2:** Interobserver agreement of categorization according to CT-FFR.

Level of observation		*n*	Cohen's kappa	95% CI	*p*
Patient		214	0.570	0.444, 0.696	<0.001
Vessel	RCA	115	0.582	0.434, 0.731	<0.001
	LAD	177	0.468	0.325, 0.611	<0.001
	LCX	114	0.230	0.052, 0.408	0.01

Interobserver agreement of categorized CT-FFR values. *P* values <0.05 were statistically significant. The threshold for hemodynamically significant CAD was ≤0.80. LAD, left anterior descending artery; LCX, circumflex artery; RCA, right coronary artery.

Observer B recategorized 14/214 patients from negative to positive and 23 patients from positive to negative, with 13 recategorizations being incorrect in regard to the standard of reference, resulting in a difference of diagnostic accuracy of Δ−6.07%. Specificity and negative predictive value (NPV) on patient level were decreased by Δ−1.82% and Δ−12.84%, respectively. On patient and vessel level, more recategorizations occurred from positive to negative than vice versa (Table 3). Gross frequency of different categorizations on vessel level was LCX: 38.6% >LAD: 23.2% >RCA: 20.9%, resulting in a net difference of Δ−3.34% in accuracy. On vessel level, specificity was slightly higher for observer B (Δ+0.73%), while other test metrics were slightly lower (Table 3). There was no discernable trend towards a differing frequency in discrepant categorizations on segment level, e.g., in the distal segments. The gross rate of discrepant categorizations into CADf^+^ or CAD^f−^on segment level is shown in Appendix 1. On segment level the difference in accuracy was Δ+1.46%.

**Table 3:** Differences in categorization and changes in diagnostic performance.

Recategorizations							Changes in diagnostic performance				
Level of observation		*n*	NN	NP	PP	PN	Δ Sen., %	Δ Spe., %	Δ PPV, %	Δ NPV, %	Δ Acc., %
QCA ≥50%	Patient	214	41	14	136	23	−10.58%	−1.82%	−4.05%	−12.84%	−6.07%
	RCA	115	43	10	48	14	−10.96%	+0.73%	−3.71%	−6.07%	−3.34%
	LM	13	12	0	0	1					
	LAD	177	36	17	100	24					
	LCX	114	34	19	36	25					
QCA ≥70%	Patient	214	41	14	136	23	−8.57%	+2.08%	−1.77%	−6.56%	−1.40%
	Vessel	419	125	46	184	64	−10.99%	+2.44%	−2.23%	−3.95%	−0.48%

(Re)categorization into hemodynamically significant CAD and differences in diagnostic performance of CT-FFR between the observer A and B. Thresholds for significant CAD were ≥50% and ≥70% diameter for QCA and ≤0.80 for CT-FFR, respectively. Acc, accuracy; LAD, left anterior descending artery; LCX, circumflex artery; LM, left main coronary artery; NN, remained negative; NP, negative-to-positive; NPV, negative predictive value; PN, positive-to-negative; PP, remained positive; PPV, positive predictive value; RCA, right coronary artery; Sen., sensitivity; Spe., specificity.

### 3.4. Standard of reference and diagnostic performance

Overall, observer B rated fewer stenoses CAD^f+^ on patient level, resulting in a lower specificity, NPV and diagnostic accuracy compared to observer A (Table 3). If the ICA cut-off were changed to ≥70% diameter lumen narrowing, the overall differences between observer A and B became much smaller (specificity: Δ+2.08 vs. Δ−1.82; NPV: Δ−6.56 vs. Δ−12.84; accuracy: Δ−1.40 vs. Δ−6.07) (Table 3).

### 3.5. Influence of image quality and coronary artery calcifications

Absolute CT-FFR values did not correlate with quantitative image quality (CNR, HU). CT-FFR values correlated weakly with qualitative image quality on patient level and in the RCA (patient: *r* = −0.116; RCA: *r* = −0.16; *p* < 0.03). CT-FFR values correlated weakly with CAC on patient level and in the LAD (patient: *r* = 0.18; LAD: *r* = 0.206; *p* < 0.009).

Categorization into CAD was independent of quantitative or qualitative image quality and of CAC (Table 4).

**Table 4:** Influence of image quality and coronary arterial calcifications.

Level of observation			*n*	Image quality									Calcium burden		
				r CNR	95% CI	*p*	r HU	95% CI	*p*	r QIQ	95% CI	*p*	r CAC	95% CI	*p*
Absolute differences	Patient		214	−0.060	−0.192, 0.075	0.385	−0.06	−0.194, 0.076	0.384	−0.116	−0.218, −0.010	0.030	0.180	0.037, 0.313	0.009
	Vessel	RCA	115	−0.038	−0.218, 0.141	0.683	−0.103	−0.270, 0.070	0.274	−0.16	−0.290, −0.021	0.030	0.094	−0.099, 0.279	0.323
		LAD	177	0.021	−0.124, 0.167	0.778	−0.046	−0.195, 0.104	0.539	−0.095	−0.199, 0.011	0.110	0.206	0.059, 0.345	0.006
		LCX	114	−0.019	−0.201, 0.168	0.837	−0.023	−0.216, 0.175	0.805	−0.059	−0.208, 0.093	0.428	0.012	−0.183, 0.205	0.901
Categorization mismatch	Patient		214	−0.003	−0.137, 0.131	0.963	0.000	−0.134, 0.134	1.000	0.060	−0.144, 0.259	0.542	−0.104	−0.235, 0.031	0.131
	Vessel	RCA	115	−0.138	−0.313, 0.047	0.142	−0.114	−0.291, 0.071	0.225	0.171	−0.088, 0.408	0.170	−0.005	−0.190, 0.180	0.955
		LAD	177	0.083	−0.065, 0.228	0.269	0.004	−0.144, 0.151	0.958	0.016	−0.184, 0.215	0.870	0.011	−0.137, 0.158	0.888
		LCX	114	0.125	−0.061, 0.302	0.187	0.091	−0.095, 0.270	0.337	−0.007	−0.144, 0.259	0.946	0.058	−0.127, 0.239	0.540

Correlation of CNR, HU, qualitative image quality and CAC with absolute CT-FFR differences between the two observers and mismatch of categorization into hemodynamically significant CAD. The first *p* value column (and 95% CI) corresponds to the influence of CNR, the second to HU, the third to QIQ while the fourth *p* value column (and 95% CI) corresponds to the influence of calcium burden. *P* values <0.05 were considered statistically significant. Thresholds for significant CAD were ≥50% diameter for QCA and ≤0.80 for CT-FFR, respectively. CNR, contrast to noise ratio; CAC, coronary artery calcium score; HU, Hounsfield unit; LAD, left anterior descending artery; LCX, circumflex artery; QIQ, qualitative image quality; RCA, right coronary artery.

## 4. Discussion

Interobserver variability of ML-based CT-FFR has not been studied extensively on a large patient cohort with a high prevalence of CAD. This study on patients before TAVR was carried out by observers with differing levels of experience and rendered somewhat different results. This led to occasional differences in categorization of hemodynamically significant CAD and moderate changes in diagnostic performance. Recategorization of patients was independent of image quality or CAC.

Absolute values of CT-FFR showed significant differences between the observers from 0.03 to 0.04 on vessel and 0.05 on patient level (Table 1). This led to occasional recategorizations into hemodynamically significant CAD when CT-FFR values fell close to the cut-off [grey zone 0.75–0.80 (28)] and likely was the most relevant reason for differences in diagnostic performance between both observers. No observer was clearly superior to the other, with observer A having higher diagnostic accuracy on patient and vessel level and observer B performing slightly better on segment level. The difference in measured values between the observers was lower for high CT-FFR values and much larger for low values. There is no clear cut-off for all levels of observation, but differences are higher below the clinical cut-off CT-FFR ≤0.80 (Figure 3). A possible explanation for this observation could be that segmentation of larger vessel lumina is easier and thus more reproducible; while the opposite is true for small vessels. This is consistent with our observation of the smallest vessel, the LCX, having the weakest interobserver agreement. The higher discrepancy of small values is of little concern, as values far below the common cut-off (0.80 or 0.75) are of little to no significance for clinical decision-making (21, 29). Absolute CT-FFR values measured by the more experienced observer (observer A) had higher variance compared to observer B (Figure 2). A possible reason for this may be a more conservative segmentation of the contrasted lumen, while observer B may have tried to extrapolate the lumen in the presence of blooming artifacts at heavily calcified lesions in a more generic way (30).

Correlation of CT-FFR values in RCA and LAD and on patient level was moderate or borderline-good. Regardless, overall correlation in our patient cohort was lower than that reported in other patient groups. Ko et al. reported a median difference of 0.03 on patient level vs. 0.05 in this study. Studies with more experienced observers reported good-to-excellent interobserver agreement (18, 19, 31). Observers with less training showed higher discrepancies and moderate agreement (32, 33). The studies consisted of much younger patient groups (60.0 ± 8.5 years; 64.6 ± 8.9 years; 61.8 ± 10.2 years; 62.7 ± 8.9 years vs. 78.9 ± 9.7 years) with fewer stenoses per patient [0.53; 1.47; 0.33; 1.22 (stenosed vessels) vs. 1.6 stenoses per patient] (18, 19, 32, 34). Median evaluation time was vastly different (Ko et al.: 27 min; Donnelly et al.: 9 min; Yang et al.: 50 min; Ihdayhid et al.: 24–38 min; current study: 24 and 28 min). In addition to the lower experience of the observers, the most likely cause for the weaker interobserver agreement in our study lies in the much different and more challenging patient group of patients prior TAVR with more frequent and perhaps higher grade and frequently calcified stenoses. Overall, more experienced observers had better agreement, while less experience only had moderate agreement between the observers (18, 32, 33). This is a direct result of differences in lumen segmentation by the user himself in ML-based CT-FFR. Resulting differences are thus no different from the reproducibility of other techniques e.g., of cCTA with good interobserver and intraobserver agreement between trained observers, and moderate agreement between untrained observers (35, 36), or even ICA interpretation (37). Other CT-FFR solutions, namely the only commercially available and FDA approved CFD-based technique has not publish data concerning observer experience and reliability. Overall, observer experience seems to have a large influence on reliable CAD diagnosis. Standardized training and certification may likely improve reliability of ML-based CT-FFR further.

Interobserver agreement of categorization of patients into hemodynamically significant CAD was similar to the correlation of absolute values with moderate and sometimes moderate-to-good correlation for the RCA, LAD and patient level. This may be reassuring as in clinical decision making most commonly a discreet cut-off is used. Despite the agreement between the observers not being optimal, it can be considered fair, taking into account the observer's differing experience (Table 2). Notably, the LCX had the lowest correlation of absolute values and lowest agreement between categorized values. Although there is no definitive answer for this observation, the LCX is generally the smallest vessel with relatively few and short segments and the second highest rate of motion during the cardiac cycle (38). This may contribute to motion- and step-artefacts consequently decreasing diagnostic performance (39) and ultimately making segmentation the most challenging in this vessel.

Observer B showed lower diagnostic performance on patient level, with especially lower sensitivity (Δ−10.58%). However, on vessel level, specificity of observer B was higher (Δ+0.73%) (Table 3). A possible explanation may be a different, more conservative segmentation approach for the less experienced observer B, e.g., when encountering artifacts. The much lower NPV on all levels of observation might be caused by observer B's lack of clinical experience, possibly leading to a generic extrapolation of the lumen in calcified lesions and failure to differentiate plaque from artifact and vice versa. Thus, more hemodynamically relevant stenoses were missed (higher false-negative count). However, it must be kept in mind that the standard of reference in this study was anatomical (ICA with QCA). The hemodynamic significance of stenosis in ICA, especially in the context of aortic valve stenosis (AS) and subsequent left ventricular (LV) hypertrophy, is unclear. Because CT-FFR is derived from the vessel cross-section and dependent on specific vessel anatomy and LV mass, AS may influence this technique and generate different values than in patients without AS and none of the resulting adaptations.

The change in diagnostic performance and specificity (Δ−1.82%) on patient level is dependent on the standard of reference. The very conservative cut-off of ≥50% for QCA might not be optimal for clinical decision-making, as it likely includes many stenoses without hemodynamic relevance. Meanwhile, CT-FFR may have classified these as not hemodynamically relevant causing false-negative categorizations. A higher ICA cut-off (e.g., QCA ≥70%) would lead to fewer false-negative categorizations by CT-FFR. Changing the standard of reference to this more stringent cut-off would decrease sensitivity, potentially increase specificity and may decrease the differences in diagnostic performance between both observers (accuracy: Δ−6.07–Δ−1.40; Table 3).

Overall, observer A likely evaluated stenoses more strictly, which explains the higher sensitivity. More clinical experience is the most probable reason for the better performance on the clinically relevant levels of observation, namely patient and vessel level. Minute differences in segmentation of the lumen may lead to different categorization into CAD whenever values fall close to the cut-off. Notably, specificity remains very similar between the observers. This can be explained by many true-negative categorizations of values relatively clearly above the cut-off. The patients that are categorized as false-negative presented CT-FFR values closer to the grey zone (0.75–0.80) than other patients (Observer A: 0.85; Observer B: 0.84). These borderline cases are prone to recategorization between both observers. As many recategorizations are correct in regard to ICA and cancel each other out, their influence on diagnostic performance is much smaller than their number leads to believe. This is supported by the number of differing CAD categorizations being larger than the actual change in diagnostic performance (recategorizations: 37/214; accuracy: Δ−6.07).

Image quality and calcium burden may interfere with the correct assessment of coronary arteries. However, the categorization into hemodynamically relevant CAD with CT-FFR was independent of CAC and image quality, which is encouraging for the use of CT-FFR in the group of patients prior to TAVR. Small, likely not clinically relevant correlations of absolute CT-FFR values and image quality and CAC were noted. Considering the number of tests performed, these findings should not be overestimated. Our findings with little to no influence of CAC on CT-FFR are consistent with the literature, with only Tesche et al. finding a degrading effect CAC on CT-FFR with very high scores (18, 19, 30, 39, 40), even though also patients with much higher CAC were included in our study. The virtual lack of correlation of CT-FFR values to image quality suggests that once a certain threshold of image quality is reached, CT-FFR may be expected to be performed reliably. Even a new deep learning algorithm for the improvement of image quality was not able to increase diagnostic performance of CT-FFR further (30).

**Figure 4:**
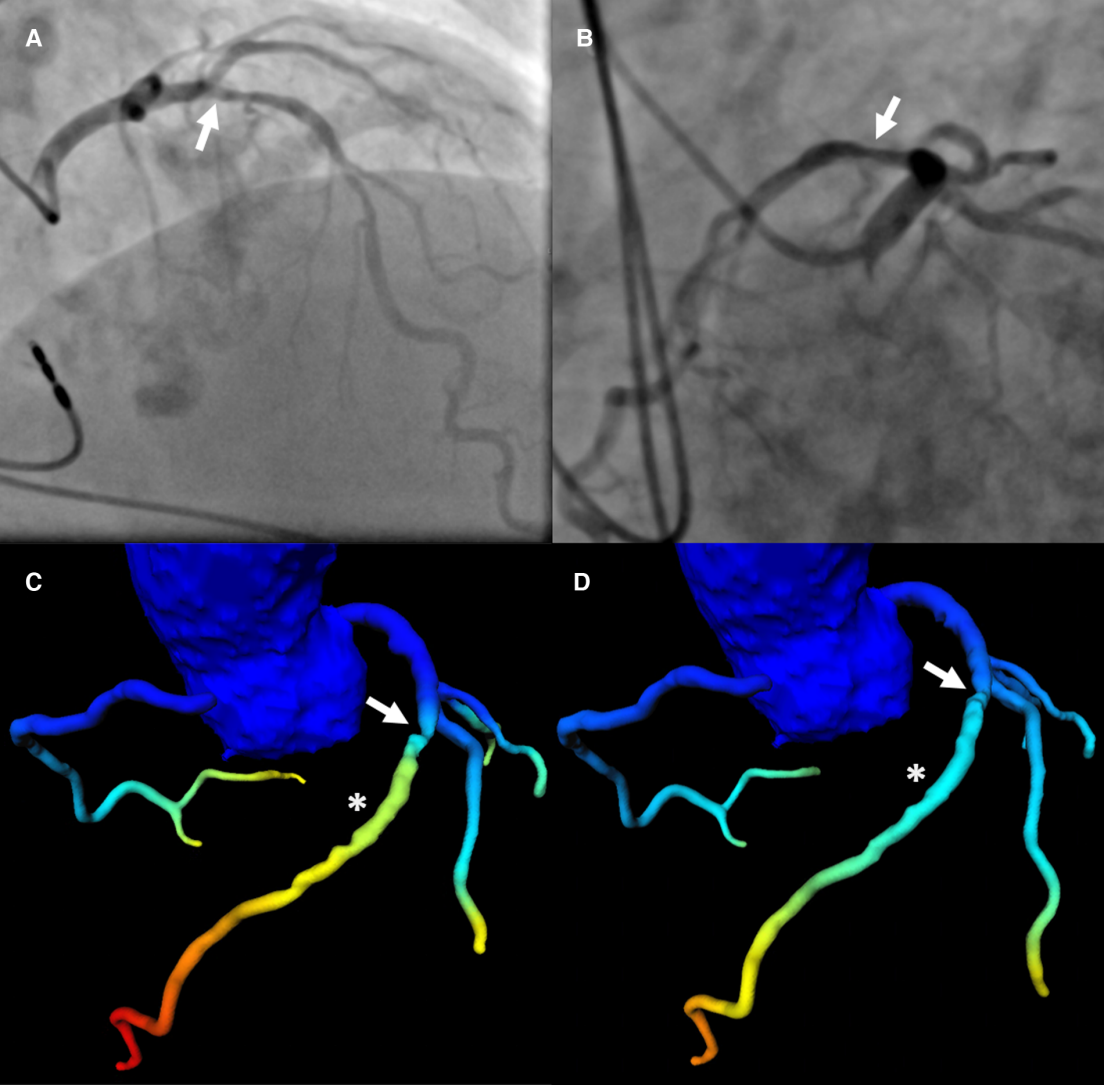
Patient with severe LAD stenosis and discrepant categorization according to CT-FFR values. Patient with severe stenosis (arrow in a-d) in the middle LAD (S7) on ICA (QCA: 78%) (**A,B**) and results of CT-FFR of observer A (**C**) and observer B (**D**) CT-FFR values were taken approximately 2 cm distal to the stenosis (asterisk in **C,D**). The CT-FFR value measured by observer A was 0.79, indicating hemodynamically significant CAD (**C**), the value measured by observer B was 0.86, indicating non-significant CAD (**D**) The threshold for hemodynamically significant CAD was ≤0.80. CAD, coronary artery disease; CT-FFR, CT-derived fractional flow reserve; ICA, invasive coronary angiography; LAD, left anterior descending artery; QCA, quantitative coronary analysis.

Patients prior to TAVR assessed with ML-based CT-FFR by two observers with differing experience were sometimes categorized differently into having hemodynamically relevant CAD or not. This was independent of image quality or CAC. This can easily be understood if values fall close to the cut-off and CT-FFR is only measured at a single point in a fixed distance distal to the stenoses (Figure 4). However, hemodynamical implications of luminal narrowing can manifest distal to that point of measurement (21). On the other hand, diffuse arteriosclerosis without a distinct stenosis may have a cumulative effect (41) additive to or independent of the stenosis measured. Instead of a single measurement with a fixed cut-off, a relative decrease of CT-FFR values along the coronary tree could perhaps prove more representative for the global hemodynamic situation (21, 41–44) of the coronary arterial vasculature.

### 4.1. Limitations

Several important limitations to our study must be noted. First, the standard of reference in this study was morphological, not functional with the conservative cut-off of ≥50% diameter on QCA. We explored how discrepancies with a more stringent cut-off would change. But ultimately, a functional standard of reference like invasive FFR would be desirable, particularly in the patient group before TAVR with hemodynamical changes, likely also in coronary artery physiology. Independently of the applied standard, the observed differences in CT-FFR values between the observers are real and likely to be similar in practical application and should be considered whenever performing CT-FFR for clinical decision making. Furthermore, patients before TAVR generally have severe AS with subsequent LV-hypertrophy. As ML-based CT-FFR also considers LV-mass in addition to vessel cross section and specific vessel anatomy for its computation, AS and underling secondary changes may influence computed CT-FFR values. Though different, clinical experience of both observer A and B was limited and may not reflect clinical practice at academic centers with dedicated experts performing such analysis, it can be assumed that expert observers are more consistent (18, 19). However, so far no data is available about the segmentation process of the commercially available off-site solution. Furthermore, the limited experience of the observers likely amplified the differences in read values, perhaps even allowing for a better evaluation of the potential disturbing factors of image quality and CAC.

## 5. Conclusion

Measurement of ML-based CT-FFR in patients prior to TAVR by observers with different clinical experience lead to discrepancies in CT-FFR values and CAD categorization, with larger discrepancies in low values and smaller discrepancies in high values. This caused a moderate difference in diagnostic accuracy. Image quality and CAC appear not to influence categorization according to CT-FFR. It seems advisable for segmentation to be performed by expert observers, particularly when values around the “grey zone” are to be expected.

## Data Availability

The raw data supporting the conclusions of this article will be made available by the authors, without undue reservation.

## Appendix

**Table 1:** Difference in CAD categorizations between observers.

Level of observation		*n*	MM (%)	95% CI (%)
Patient		214	17.3	12.8, 22.9
Vessel	RCA	115	20.9	14.4, 29.2
	LM	13	7.7	1.4, 33.3
	LAD	177	23.2	17.6, 29.9
	LCX	114	38.6	30.2, 47.8
Segment	1	59	23.7	14.7, 36.0
	2	59	20.3	12.0, 32.3
	3	42	28.6	17.2, 43.6
	4	18	5.6	1.0, 25.8
	16	8	25.0	7.1, 59.1
	5	13	7.7	1.4, 33.3
	6	56	25.0	15.5, 37.7
	7	20	45.0	25.8, 65.8
	8	123	28.5	21.2, 37.0
	9	42	19.0	10.0, 33.0
	10	53	41.5	29.3, 54.9
	17	22	31.8	16.4, 52.7
	11	62	40.3	29.0, 52.7
	12	50	40.0	27.6, 53.8
	13	33	36.4	22.2, 53.4
	14	12	33.3	13.8, 60.9
	15	1	0.0	0.0, 79.3
	18	0	–	–

Rate of recategorization on patient, vessel and segment level and corresponding 95% CI. CI, confidence interval; LAD, left anterior descending artery; LM, left main coronary artery; LCX, circumflex artery; MM, mismatched coronary artery disease categorization; RCA, right coronary artery.
